# Idiopathic Gingival Fibromatosis

**DOI:** 10.5005/jp-journals-10005-1086

**Published:** 2011-04-15

**Authors:** Prathibha Anand Nayak, Ullal Anand Nayak, Vishal Khandelwal, Nupur Ninave

**Affiliations:** 1 Reader, Department of Periodontics, Modern Dental College and Research Center, Airport Road, Gandhi Nagar Indore, Madhya Pradesh, India; 2 Professor, Department of Pedodontics and Preventive Dentistry, Modern Dental College and Research Center, Airport Road Gandhi Nagar, Indore, Madhya Pradesh, India; 3 Senior Lecturer, Department of Pedodontics and Preventive Dentistry, Modern Dental College and Research Center Gandhi Nagar, Indore, Madhya Pradesh, India; 4 Senior Lecturer, Department of Pedodontics and Preventive Dentistry, USPM Dental College and Research Center Nagpur, Maharashtra, India

**Keywords:** Idiopathic gingival fibromatosis, Gingival hyperplasia.

## Abstract

Idiopathic gingival fibromatosis is a rare heriditary condition characterized by slowly progressive, nonhemorrhagic, fibrous enlargement of maxillary and mandibular keratinized gingiva caused by increase in submucosal connective tissue elements. This case report gives an overview of gingival fibromatosis in a 11-year-old female patient who presented with generalized gingival enlargement. Based on the history and clinical examination, the diagnosis was made and the enlarged tissue was surgically removed. The patient was being regularly monitored clinically for improvement in her periodontal condition as well as for any recurrence of gingival overgrowth.

## INTRODUCTION

Idiopathic gingival fibromatosis (IGF) is an uncommon, benign, hereditary condition with no specific cause. IGF is characterized by a slowly progressive, nonhemorrhagic, fibrous enlargement of maxillary and mandibular keratinized gingiva. It occurs either as an isolated disease or combined with some rare syndromes or chromosome disorders. According to Gorlin et al, IGF is most commonly associated with hypertrichosis, also occasionally associated with mental retardation and epilepsy. Syndromes that have been associated with IGF are Zimmerman-Laband syndrome (defects of bone, nail, ear and nose accompanied by splenomegaly), Murray-Puretic-Drescher syndrome (multiple dental hyaline tumors), Rutherford syndrome (corneal dystrophy), Cowden syndrome (multiple hamartomas), and Cross syndrome (hypopigmentation with athetosis).^1^ More recently, hearing loss and supernumerary teeth have been reported to be associated with HGF. The condition has also been reported in association with deficiency of growth hormone caused by lack of growth hormone release factor.^2^

Synonyms of IGF include hereditary gingival fibro-matosis, elephantiasis gingivae, congenital hypertrophy of gingiva, fibromatosis gingivae, congenital macrogingivae and hypertrophic gingiva.

The hyperplastic gingival tissue is usually pale-pink, firm, has leathery consistency and presents a characteristic pebbled surface. Exaggerated stippling may be present. The enlarged tissues may partially or totally cover the dental crowns, can cause diastemas, pseudo-pocketing, delay or impede tooth eruption and can aggressive periodontitis due to poor oral hygiene. In severe cases, it may lead to mastication and speech impediments or lip closure difficulties. The condition may be painful when tissues are traumatized during mastication. The condition may present as a nodular form characterized by the presence of multiple tumors in the interdental papillae or a more common symmetric form resulting in uniform enlargement of gingiva or a combination of both.^3^ It may be unilateral or bilateral, localized or generalized and can affect both maxilla and mandible. The disease commences frequently when deciduous or permanent teeth begin to erupt. Females and males appear to be equally affected.

Histologically, the affected tissues are generally composed of dense connective tissue rich in coarse collagen fibers and are highly differentiated with young fibroblasts and scarce blood vessels. The epithelium is hyperkeratotic with elongated rete pegs. Unusual findings include the presence of small calcified particles, amyloid deposits, islands of odontogenic epithelium and osseous metaplasia in the connective tissue and ulcerations of the overlying mucosa.^4^

HGF is genetically heterogeneous and can occur in either autosomal dominant (common) or recessive forms. Families are affected across the generations and a positive family history is always present. In IGF, no causative agent can be identified and a family history is lacking. If the inheritance is autosomal dominant, then the phenotypic frequency is 1 in 750,000 people and the gene frequency is 1 in 350,000.^5^

## CASE REPORT

**Fig. 1:**
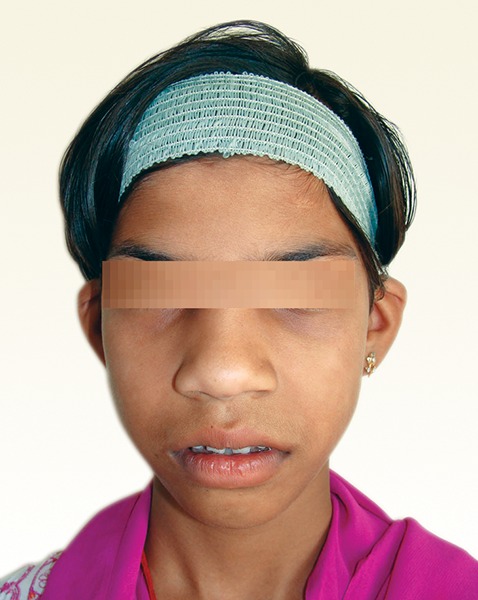
Preoperative photograph of the patient

A 11-year-old girl, accompanied by her parents reported with the complaint of swollen gums. The patient presented with gradual and progressive enlargement of both upper and lower gingival tissues from the age of 6 years. The enlargement had led to incompetent lips, poor esthetics and also hindered in speech and mastication. She appeared apprehensive and lacked confidence due to gummy smile (Fig. 1).

The family and postnatal histories were noncontributory. The patient exhibited no signs of hypertrichosis or mental retardation and had no history of epilepsy or intake of medication known to cause gingival overgrowth.

**Fig. 2:**
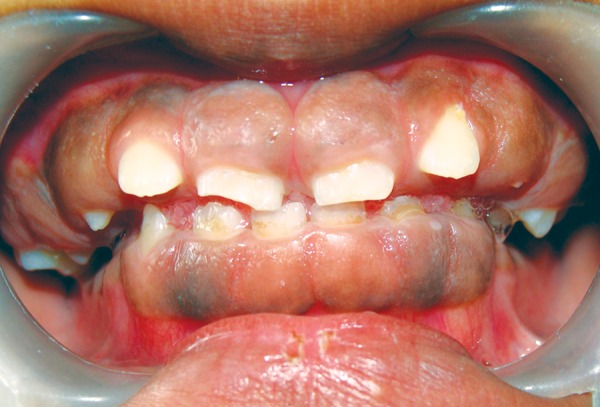
Gingival overgrowth in anterior teeth region

**Fig. 3:**
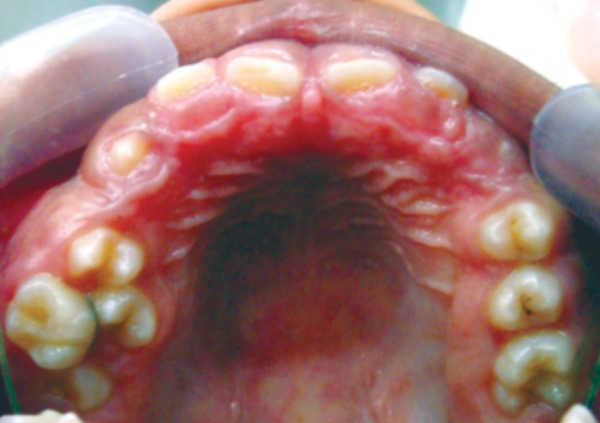
Gingival overgrowth in upper arch

**Fig. 4:**
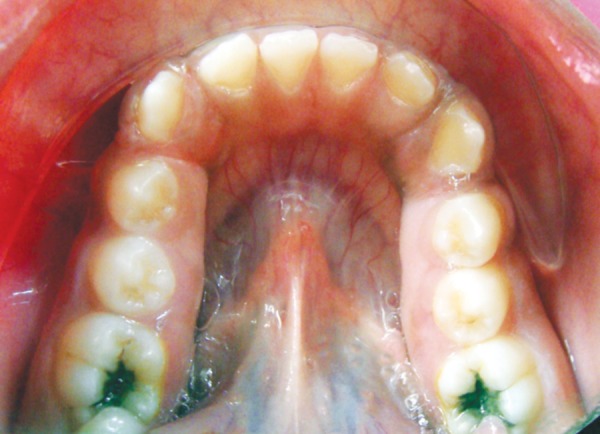
Gingival overgrowth in lower arch

On examination, patient had bilaterally symmetrical face with incompetent lips and convex profile. An intraoral examination revealed generalized, smooth enlargement of the gingiva involving upper and lower arches (Figs 2 to 4). The gingiva was pink in color with superimposed melanin pigmentation. The consistency was firm and stippling was present. The enlarged gingiva covered the crowns of all the teeth till the incisal or occlusal third region. Generalized pseudo-pockets were observed with no bleeding on probing. In the posterior region, scissor bite was present due to which gingiva was traumatized during mastication.

The dentition revealed the presence of all permanent teeth (except second and third molars) and upper right second primary molar (tooth 54), which was buccally placed and physiologically mobile.

An orthopantomogram was advised, which was non-contributory. Other investigations performed were complete hemogram, routine urine analysis and thyroid tests (T3, T4, TSH). All the reports were within the physiological limits. The alginate impressions of upper and lower arch were made and study models were obtained for the record purpose.

With the available data regarding history and clinical features, a provisional diagnosis of idiopathic gingival fibromatosis was arrived at. The parents of the patient were considering orthodontic treatment for the correction of malaligned teeth but the amount of crown structure exposed on most teeth were insufficient to allow accurate placement of brackets.

Accordingly, a treatment plan was formulated which constituted quadrant-wise gingivectomy along with extraction of tooth 54, followed by restorative and fixed orthodontic procedures. The treatment procedure was explained to the patients and parent, and accordingly written consent was obtained. As the patient was cooperative, gingivectomy was planned under local anesthesia containing 2% lignocaine with 1:200000 epinephrine. An external bevel incision was given and the gingiva was excised till the desired crown lengthening was achieved. After the surgery, the site was irrigated with betadine and a Coe-pak (non-eugenol, hard and fast set) was given for seven days. The patient was advised to take analgesics and rinse twice daily with 0.2% Chlorhexidine mouthwash for two weeks.

**Fig. 5:**
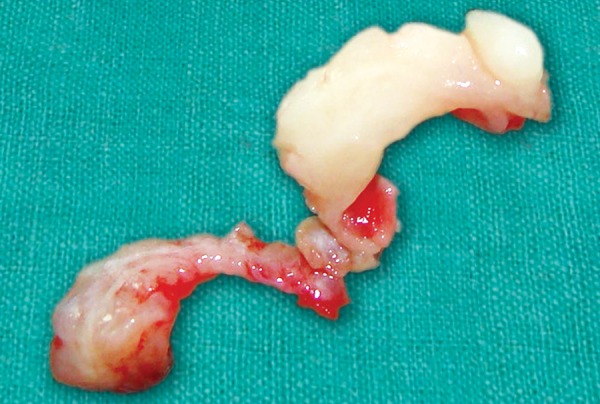
Excised gingival tissue

**Fig. 6:**
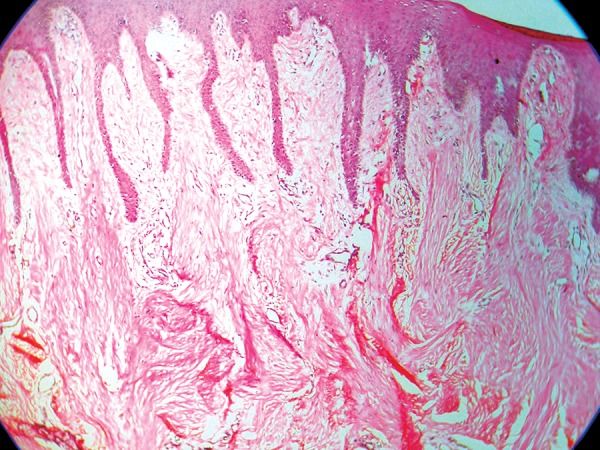
Histopathological specimen showing stratified squamous epithelium with long slender rete pegs and connective tissue with dense collagen stroma

The excised gingival tissue was sent for the histopathological evaluation (Fig. 5). The H&E staining of the specimen revealed stratified squamous epithelium with focal keratinization, long slender rete pegs and the subepithelium showed dense collagen stroma in connective tissue (Fig. 6). These findings led to the final diagnosis of generalised idiopathic gingival fibromatosis.

**Fig. 7:**
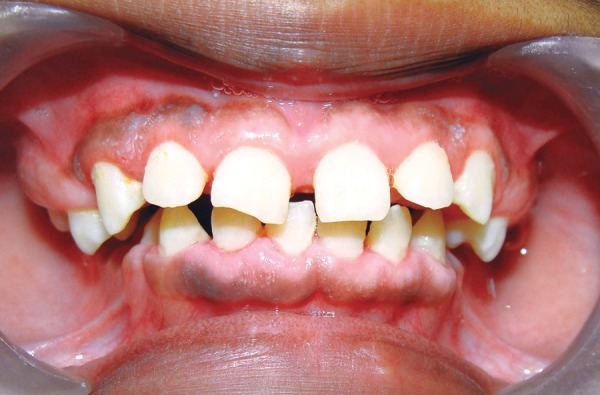
Postoperative - increased clinical crown length

**Fig. 8:**
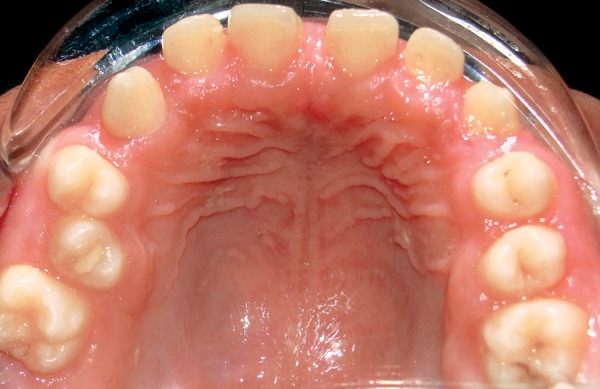
Postoperative - upper arch

**Fig. 9:**
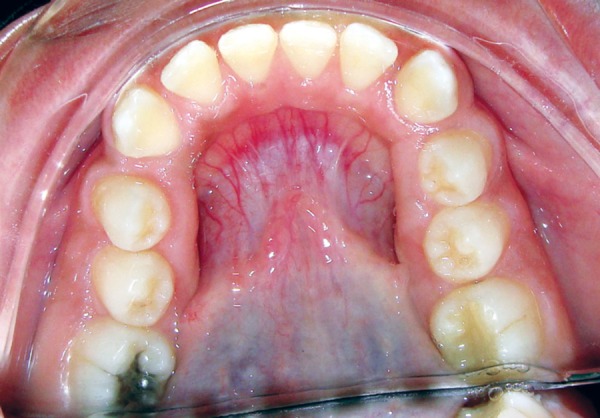
Postoperative - lower arch

**Fig. 10:**
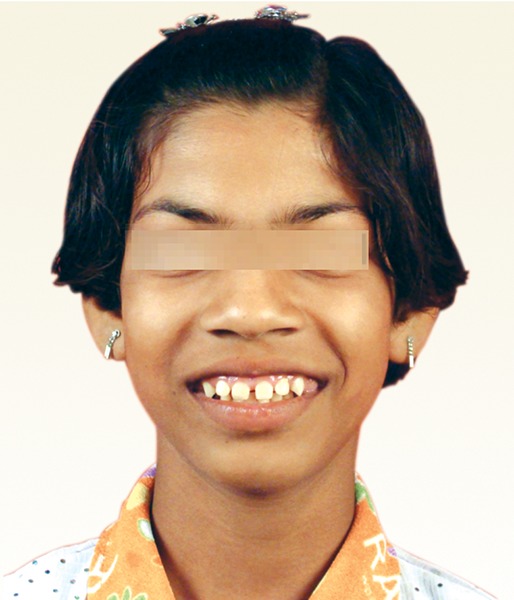
Postoperative photograph of the patient

The periodontal dressing was removed after a week and healing was found to be satisfactory (Figs 7 to 9). The patient was then placed on a schedule of periodic recall visits for maintenance care (every three months). The oral hygiene maintenance was reinforced at every recall. No recurrence of gingival enlargement was observed one year after the surgery (Fig. 10). The patient will be undergoing orthodontic treatment for the correction of malaligned teeth.

## DISCUSSION

Gingival fibromatosis can occur as an isolated condition or be associated with other diseases or syndromes and can be localized or generalized. The different forms can be classified as following:^6^

1. Isolated HGF
2. Isolated IGF
3. GF with hypertrichosis
4. GF with hypertrichosis and mental retardation and/or epilepsy
5. GF with mental retardation and/or epilepsy
6. GF associated with other diseases as part of a syndrome.

The noncontributory histories (family, medical, postnatal and drug histories) and the clinical appearance of bilateral uniform enlargement of gingiva led to the diagnosis of a symmetric form of generalized isolated idiopathic gingival fibromatosis in the present case. The generalized form of isolated IGF is more common in male patients in contrast to the present case, while the localized type occurs in the female patients. Hereditary HGF tends to occur more frequently as a generalized type than IGF. The ratios of generalized to localized types in HGF and IGF have been reported as 15.2:1 and 1.6:1 respectively.^7^

IGF is a slowly progressive, benign enlargement that affects the attached gingiva as well as gingival margin and interdental papillae unlike drug-induced enlargement, which is limited to gingival margin and interdental papillae. Why the HGF phenotype appears localized to the gingiva is unknown. The keratinized masticatory mucosa is recognized to be developmentally unique and different tissue specific signaling pathways in this unique tissue may be responsible for the limited tissue distribution of the gingival fibromatosis phenotype.^8^ Severity may vary from mild involvement of one quadrant to severe involvement of all four quadrants and can even distort the appearance of jaws.

Though the cause of IGF is unknown, this appears to be a genetic predisposition. The condition may manifest as an autosomal dominant or less commonly as autosomal recessive mode of inheritance. Autosomal dominant non-syndromic forms have been genetically linked to the chromosome 2p21-p22 and 5q13-q22.^9,10^ It is possible that isolated GF may result from a single gene mutation while syndromic forms may result from alterations of multiple genes or perhaps a gene dosage effect.^11^ Recently, a mutation in son of sevenless-1(SOS-1) gene has been held responsible for this rare hereditary condition. SOS-1 gene codes for a protein that activates the ras pathway, which signals cell growth. When the gene is not mutated, it is involved in the growth of normal, healthy gums. When mutated, it results in gingival fibromatosis. A recent report presented evidence that a single nucleotide-insertion mutation in codon 1083 of the SOS-1 gene localized to chromosome 2p21-p22 is the cause of HGF in humans.^8^ However, HGF displays genetic heterogeneity and mutations in other genes are also likely possible.

Histologically, the gingival hyperplasia is mainly due to an increase and thickening of collagen bundles in connective tissue stroma. The nodular appearance can be attributed to the thickened hyperparakeratinized epithelium. The cellular and molecular mechanisms that lead to this condition are not well understood. HGF keratinocytes seem to have an important role in pathogenesis by inducing extracellular matrix accumulation by fibroblasts.^12^ According to a recent report, increased proliferation and elevated production of extracellular matrix molecules, type I collagen and fibronectin could contribute to the clinically increased bulk of gingiva.^13^ Several authors suggest that more the fibroblasts present, greater the chance for recurrence.^14^

Enlargement usually begins with the eruption of deciduous or permanent dentition; it may rarely present at birth or arise in adulthood. The most extensive enlargement appears to occur during loss of deciduous teeth or in early stages of eruption of permanent teeth. It progresses rapidly during active eruption and decreases with the end of this stage.^15^ The continuing recurrence of the enlargement following surgery and a permanent remolding of tissue after extraction of teeth suggests the importance of the presence of teeth and the environment of gingival crevice in the pathogenesis of GF.^16^ Emerson recommended that the best time for the excision of gingival enlargement is when all the permanent teeth have erupted.^17^

Various procedures available for removal of GF include surgery, electrocautery and use of a carbon dioxide laser. If carbon dioxide laser is not available, the most effective method for removing large quantities of gingival tissue especially when there is no attachment loss and all the pocketing is false is the conventional, external bevel gingivectomy.^18^ In the case presented here, quadrant-wise gingivectomy was performed with periodontal pack placement for one week and 0.2% Chlorhexidine rinse twice a day for two weeks after each surgery.

Our patient is being regularly monitored clinically for improvement in her periodontal condition, as well as for any recurrence of gingival overgrowth. She will be undergoing orthodontic treatment for the correction of malaligned teeth. It is conceivable that orthodontic treatment might stimulate recurrence in some patients by impeding periodontal hygiene maintenance and thereby necessitating repeat surgery following it. However, after orthodontic therapy, proper tooth repositioning and lip seal prevent mouthbreathing, which might otherwise exacerbate the condition.
